# Correlation of myocardial strain by CMR-feature tracking with substrate abnormalities detected by electro-anatomical mapping in patients with nonischemic cardiomyopathy

**DOI:** 10.1007/s10840-023-01553-5

**Published:** 2023-05-02

**Authors:** Deep Chandh Raja, Indira Samarawickrema, Jaganaathan Raman Srinivasan, SaratKrishna Menon, Souvik Kumar Das, Sanjiv Jain, Lukah Q. Tuan, Benoit Desjardins, Francis E. Marchlinski, Walter P. Abhayaratna, Prashanthan Sanders, Rajeev K Pathak

**Affiliations:** 1 ANU School of Medicine and Psychology, Australian National University, 54 Mills Road, Acton, 2601 ACT Australia; 2 University of Canberra, Canberra, Australia; 3 Canberra Health Services, 2 Garran place, Garran, Canberra, 2605 Australia; 4 University of Newcastle, Newcastle, NSW Australia; 5 Canberra Heart Rhythm, 2 Garran Place, Garran, 2605 Australia; 6 Electrophysiology Section, Hospital of the University of Pennsylvania, Philadelphia, USA; 7 Center for Heart Rhythm Disorders, University of Adelaide and Royal Adelaide Hospital, Adelaide, Australia

**Keywords:** Electroanatomical mapping, Cardiac magnetic resonance imaging, Myocardial strain, Circumferential strain, Longitudinal strain, Low voltage zone

## Abstract

**Background:**

Late gadolinium enhancement (LGE) detected by cardiac MRI (CMR) has low correlation with low voltage zones (LVZs) detected by electroanatomical mapping (EAM). We aim to study correlation of myocardial strain by CMR- Feature Tracking (FT) alongside LGE with LVZs detected by EAM.

**Methods:**

Nineteen consecutive CMRs of patients with EAM were analyzed offline by CMR-FT. Peak value of circumferential strain (CS), longitudinal strain (LS), and LGE was measured in each segment of the left ventricle (17-segment model). The percentage of myocardial segments with CS and LS > −17% was determined. Percentage area of LGE-scar was calculated. Global and segment–wise bipolar and unipolar voltage was collected. Percentage area of bipolar LVZ (<1.5 mV) and unipolar LVZ (<8.3 mV) was calculated.

**Results:**

Mean age was 62±11 years. Mean LVEF was 37±13%. Mean global CS was −11.8±5%. Mean global LS was −11.2±4%. LGE-scar was noted in 74% of the patients. Mean percentage area of LGE-scar was 5%. There was significant correlation between percentage abnormality detected by LS with percentage bipolar LVZ (*r* = +0.5, *p* = 0.03) and combined percentage CS+LS abnormality with percentage unipolar LVZ (*r* = +0.5, *p* = 0.02). Per-unit increase in CS increased the percentage area of unipolar LVZ by 2.09 (*p* = 0.07) and per-unit increase in LS increased the percentage area of unipolar LVZ by 2.49 (*p* = 0.06). The concordance rates between CS and LS to localize segments with bipolar/unipolar LVZ were 79% and 95% compared to 63% with LGE.

**Conclusions:**

Myocardial strain detected by CMR-FT has a better correlation with electrical low voltage zones than the conventional LGE.

**Supplementary Information:**

The online version contains supplementary material available at 10.1007/s10840-023-01553-5.

## Introduction

Cardiac magnetic resonance (CMR) imaging is one of the most important diagnostic tools for characterization of myocardial diseases [1]. Late gadolinium enhancement (LGE) detected by CMR has shown to predict ventricular arrhythmic events in dilated non-ischemic cardiomyopathy (NICM) [2]. However, the correlation of LGE with substrate abnormalities detected by invasive electroanatomical mapping (EAM) is modest in NICM [3]. Studies have noted that 52% of EAM-scars in the RV and 36% of EAM-scars in the LV can be missed by LGE [3, 4]. This is mainly because of the inability of LGE to detect the complex scar patterns in NICM.

Novel CMR parameters such as T1 mapping have recently been shown to be better predictors of the intramural concealed fibrosis seen in NICM [5]. Myocardial deformation parameters studied by CMR-strain imaging have the ability to detect deep intramural mechanical abnormalities within the myocardium of the left ventricle (LV) [6]. Moreover, CMR-strain is a reproducible measure which has been shown to have strong association with clinical outcomes such as death and SCD in dilated CMP [7]. However, CMR-strain has never been correlated with electrical abnormalities detected by EAM. In this study, we sought to investigate the correlation of global and segment-wise CMR-strain parameters along with the LGE-scar analysis with global and segment-wise low voltage zones picked up by EAM.

## Methods

Our study population consisted of consecutive patients with NICM enrolled prospectively for CMR studies presenting to the Canberra Heart Rhythm Centre, in the period between November 2019 and November 2021. The patients underwent invasive electrophysiological studies with voltage mapping and radiofrequency ablation when deemed necessary. The diagnosis of NICM was made with corroborative evidence of LV dysfunction (LV ejection fraction <50%), in the absence of significant coronary artery disease (>50% stenosis as assessed by coronary angiography). The following categories of NICM were excluded: congenital heart diseases; hypertrophic cardiomyopathy; arrhythmogenic right ventricular cardiomyopathy (ARVC); and LV noncompaction. In addition, pacing-dependent patients, persistent/long standing atrial fibrillation patients were excluded for the study. The study was conducted as per the ethical guidelines of the Declaration of Helsinki and was approved by the Human Research and Ethics Committee of The Canberra Hospitals (2019/ETH13256).

### CMR acquisition protocol

CMR was performed on a 1.5 T scanner (Ingenia; Philips Healthcare, the Netherlands) with a cardiac phased-array receiver surface coil and electrocardiogram (ECG) gating. For the assessment of LV functions, cine imaging was performed by using a steady-state free precession (SSFP) sequence in the vertical long axis, horizontal long axis and short axis. In patients with devices, Turbo Spin echo for cine imaging was used when significant susceptibility artifact was present on SSFP imaging. Patients with significant artifacts due to devices were excluded from the study. Pacing-dependent patients were excluded from the study. In patients with cardiac resynchronization devices, biventricular pacing was on. Standard parameters were repetition time/echo time 3.6/1.8 ms; sense factor 2, flip angle — 60°; section thickness — 8 mm; field of view — 300 mm. For scar assessment, LGE images covering the entire LV were acquired approximately 15 min after an intravenous injection of 0.2 mmol/kg gadobenate dimeglumine contrast agent. The LGE-images were acquired using a magnitude inversion-recovery (IR) or phase sensitive inversion recovery (PSIR) gradient–recalled echo sequence with 8.0 mm slice thickness. A wideband LGE sequence was used to minimize artifacts from the battery pack in subjects with device implants.

### Myocardial strain and LGE-scar assessment by CMR

All the CMR studies were analysed offline by using a dedicated software called the Segment Medviso version 3.3 RX [8] (www.medviso.com/segment). The base and the apex of the LV were defined from the short-axis slices. The endocardial and epicardial borders were traced manually in the short axis, apical 3-chamber long-axis, apical 2-chamber long-axis and apical 4-chamber long-axis images in end-diastole and end-systole. Adequate precaution was exercised to avoid blood pool contamination and to exclude the papillary muscles. CMR studies with poor image quality or missing slices were excluded from the analysis. Segmentation of the LV into the standard 17-segments was carried out by the software [9]. LV dimensions, volume, mass and LVEF were estimated automatically. Myocardial strain was measured throughout the cardiac cycle by myocardial Feature-Tracking (FT) [10]. The peak measurements of three strain parameters — circumferential strain, radial strain, and longitudinal strain — were considered for analysis. Global and segment-wise strain values were extracted from the software. Circumferential strain and radial strain were measured from 16 segments (excluding apical segment). Longitudinal strain was measured from all 17 segments. The strain measurements were performed in a blinded fashion by two experienced analysts.

Inter-observer reproducibility of all the three strain parameters was studied. Due to high inter-observer variability with radial strain, only circumferential strain and longitudinal strain were considered for localization of abnormalities in each segment. Peak circumferential strain and longitudinal strain values of >−17% were considered abnormal. This cut-off was chosen arbitrarily based on multiple studies reporting outcomes with the same cut-offs [7, 11-13]. However, there are no existing large-scale studies which have validated abnormal segmental strain values. The number of abnormal segments were counted for circumferential strain, longitudinal strain and composite of circumferential strain and longitudinal strain (circumferential + longitudinal strain). Percentage abnormal myocardium was derived as a proportion of abnormal segments. LGE-scar was determined in each segment by the EWA-algorithm [14]. Automatic delineation of scar borders was performed and was verified by an experienced CMR-analyst. The percentage area of LGE-scar for the total LV and in each segment was extracted from the software. Segmental circumferential strain, longitudinal strain and percentage area of LGE-scar was displayed on a 17-segment color polar plots.

### Electro-anatomical mapping and catheter ablation

A systematic protocol for EAM was followed uniformly in all patients. All antiarrhythmic drugs were discontinued routinely at least 5 half-lives before the procedure. Three-dimensional (3D) left ventricular geometry was reconstructed by intracardiac echocardiography (ICE; 64-element, 5.5 to 10 Hz; SOUNDSTAR^TM^, CARTOSOUND^TM^ module Biosense Webster, La Jolla, CA, USA). EAM of the endocardial LV was performed using the CARTO 3 Version 7 mapping system (Biosense Webster) using a multi-electrode mapping catheter (PENATRAY^TM^, Biosense Webster). The geometry created using ICE was registered to an endocardial 3D shell of LV acquired by the mapping catheter. High density mapping of the LV was performed at all the segments of the LV. The low voltage zones were addressed further by point-by-point mapping using a deflectable 3.5-mm irrigated-tip mapping catheter with contact force (THERMOCOOL SMARTTOUCH-SF^TM^, Biosense Webster) during sinus rhythm. In patients with cardiac resynchronization devices, biventricular pacing was on. Geometry, bipolar and unipolar electrograms (EGMs) were simultaneously recorded and all segments of the ventricle were sampled. The mitral and aortic annuli were defined by ICE. In addition, the mitral annulus was verified as that with a 1:1 ratio between atrial and ventricular electrograms. Low voltage points acquired with <3 g contact force, <10 mm from the endocardial shell, points with unstable cycle length, points within 1 cm of the aortic and mitral valve annulus were all excluded from analysis. Bipolar signals were filtered at 30 to 400 Hz. Unipolar signals were measured between the tip electrode and Wilson-central terminal and were filtered at 1 to 240 Hz. The fill threshold was set to 10 mm.

Ventricular tachycardia (VT) induction was attempted in all patients with programmed ventricular stimulation with triple extrastimuli from at least two right ventricular or LV sites with at least two drive cycle lengths. Induced VTs were identified as clinical if they matched the cycle length and morphology of the stored electrograms from the ICD or the 12-lead ECG when available. VT entrainment was performed if the VTs were hemodynamically stable. Pace mapping at threshold was performed to match the inducible VT, in case the VT was hemodynamically unstable or non-sustained and repetitive. Substrate modification was performed at the regions of good pace map, aiming at elimination of local abnormal ventricular activity potentials, late potentials, and low amplitude fractionated electrograms. The contact force catheter was also used for ablation. The primary endpoint for ablation was elimination of the clinical VT and monomorphic nonclinical VT.

### Segmental analysis of EAM data

The raw EAM datasets were exported from the CARTO system and imported into an EP Lab Research Works application (www.eplabworks.com). Automatic annotation of all EGMs and automatic segmentation of the LV into 17 segments were performed. The landmarks for LV segmentation were set in cooperation with 2 experienced electrophysiologists. The EGM analysis was performed in each segment. Annotation of all electrograms were individually reviewed. Low voltage and scar regions were defined based on standard abnormal values for bipolar low voltage zones (Bi-LVZ; <1.5 mV), bipolar scar (Bi-Scar; <0.5 mV) and unipolar low voltage zone (Uni-LVZ; <8.3 mV) [15, 16]. The bipolar and unipolar low voltage maps were displayed on 17-segment color polar plots. The extent of the low voltage zones in the entire endocardial surface as well as in each segment was quantified as endocardial surface area (cm^2^) and proportion (%) of abnormal to the total LV endocardial surface area and proportion (%) of the abnormal area within each segment.

### Clinical follow-up

The duration of follow-up was calculated from the time of the EAM study. The patients were followed up every 6 months at the heart failure out-patient clinic, or earlier if symptomatic. All patients with device implants were followed up with remote monitoring-based device interrogations every month. Device therapies were reviewed for appropriateness by experienced cardiac electrophysiologists.

### Statistical analysis

Continuous variables which were normally distributed were expressed as mean±SD. Categorical variables were presented as proportions in percentages. The inter-observer variability was assessed with Bland-Altman analysis, coefficient of variation (CV) and the 95% limits of agreement (LOA) were studied for the dispersion around the mean. The correlation was tested by Spearman’s rank coefficients for parameters detected by CMR and % bipolar LVZs and by Pearson’s correlation for parameters detected by CMR and % unipolar LVZs. Correlation values were ranked as mild (0–0.3), moderate (0.4–0.6) and high (0.7–1.0). Linear regression models were studied for each variable, to determine the increase of LVZ per-unit increase in the independent variable. Student-*t* test was carried out to identify the differences in the mean LVZ in the patients with and without VT. The 17-segment bull’s eye maps of the CMR circumferential strain, CMR longitudinal strain, CMR LGE, bipolar voltage EAM and unipolar voltage EAM were used for side-by-side comparisons. The segmental abnormalities detected by LGE, abnormal circumferential strain and abnormal longitudinal strain in each patient were classified as concordant with EAM, if the segments also had bipolar/unipolar voltage abnormalities. The concordance rates were thus presented as proportion of correct classifications. Differences, correlation-coefficients, and the odds ratio were considered statistically significant at the two-sided *p* < 0.05 level. All the analyses were performed using STATA 17.0 (STATA Corporation, Texas, USA).

## Results

### Study recruitment and patient characteristics

In the period from November 2019 to November 2021, 44 patients of NICM who underwent CMRs, were screened for enrolment. Of these, based on the exclusion criteria for the study, the following category of NICM patients (*n* = 25) were excluded: pacing-dependent (*n* = 6); persistent atrial fibrillation (*n* = 4); hypertrophic cardiomyopathy (*n* = 4); ARVC (*n* = 3); non-compaction (*n* = 2); congenital heart disease (*n* = 2). Due to artifacts from existing leads in patients with device implants resulting in poor quality of the CMR images, 4 patients were excluded for the offline analysis.

The characteristics of the 19 patients included in the study are summarized in Table 1. The mean age was 61.8±11 years. Fifty eight percent were male. The categories of NICM patients included in the analysis were: idiopathic dilated cardiomyopathy (*n* = 10); presumed sarcoidosis based on clinical in addition to findings on CMR and PET-CT scan imaging (*n* = 4); PVC cardiomyopathy with 24-h PVC burden >20% (*n* = 4); alcoholic cardiomyopathy (*n* = 1). Diabetes was prevalent in 21% and hypertension in 26% of patients. Among the heart failure medications, 84% were on betablockers, 68% on neprilysin/angiotensin converting enzyme inhibitor/angiotensin receptor blockers; 32% on aldosterone blockers. In addition to betablockers, amiodarone was used in 4 patients and mexiletine was used in 2 patients with a history of VT. The mean LVEF was 37.4±13%. LVEF <35% was noted in 42% of the patients. Ablation was performed in 58% of the patients, defibrillator was implanted in 53% and cardiac resynchronization devices were implanted in 26% of the patients.

**Table 1:** Demographic and clinical variables in patients with nonischemic cardiomyopathy

Variable (*n* = 19)	Frequency (Proportion)	Variable (*n* = 19)	Frequency (Proportion)
Age, years	61.8±11.2	Diabetes	4 (21.1%)
Men	11 (57.9%)	Hypertension	5 (26.3%)
Chest pain	2 (10.5%)	Mean EDD (mm)	48±14
Dyspnoea	13 (68.4%)	Mean ESD (mm)	32±9
Syncope	4 (21.1%)	Mean LVEF, %	37.4±12.7
Duration of symptoms, months	13.1±12.4	LVEF <35%	8 (42.1%)
NYHA I	4 (21.1%)	Hb, g/L	143.9±13.4
NYHA II	12 (63.2%)	Creatinine, μmol/L	85.5±20.9
NYHA III	3 (15.8%)	eGFR, ml/min/m [2]	74.8±13.6
History of AF	3 (15.8%)	ICD	10 (52.6%)
History of VT	11 (57.9%)	CRTd	5 (26.3%)
Inducible VT	16 (84%)	Beta blockers	16 (84.2%)
VT ablation	16 (84%)	ARNi/ACEi/ARB	13 (68.4%)
Serum BNP (pg/ml)	158±126	Spironolactone	6 (31.6%)
QRS>120 ms (LBBB)	5 (26.3%)	Amiodarone/Mexiletene	4 (21%)/ 2 (11%)
QRS≤120 ms	14 (74%)	Failed antiarrhythmic drugs	6 (32%)

Abbreviations: *SD*, standard deviation; *Hb*, haemoglobin; *eGFR*, estimated glomerular filtration rate; *AF*, atrial fibrillation; *VT*, ventricular tachycardia; *BNP*, brain natriuretic peptide; *LBBB*, left bundle branch block; *EDD*, end-diastolic diameter; *ESD*, end-systolic diameter; *LVEF*, LV ejection fraction; *Hb*, hemoglobin; *eGFR*, estimated glomerular filtration rate; *ICD*, implantable cardiac defibrillators; *CRTd*, cardiac resynchronization therapy device; *ARNi*, angiotensin receptor and neprilysin inhibitor; *ACE*, angiotensin-converting enzyme; *ARB*, angiotensin II receptor blocker

### CMR-strain, LGE-scar and EAM characteristics (Table 2)

Mean peak global circumferential strain was −11.8±4.5%, mean peak global radial strain was +22.4±8.7% and mean peak longitudinal strain was −11.2±3.8%. Among the three measures of strain, the inter-observer variability was highest with peak radial strain (LOA −37.9–8.1; CV 17.3%) followed by peak longitudinal strain (LOA 2.2–19; CV 9.3%) then peak circumferential strain (LOA 2.4–19.9; CV 5.4%). Hence, peak longitudinal and peak circumferential strain was considered for further analysis. Percentage segmental abnormalities detected were 75±20% with circumferential strain, 70±16% with longitudinal strain and 54±20% with the composite of circumferential + longitudinal strain. LGE-scar was detected in 63% of the patients (*n* = 12), of whom 75% had patchy scar in a single focus and 25% had multifocal scar. Among the 7 patients with no LGE-scar, 5 patients had idiopathic dilated cardiomyopathy and 2 patients had presumed PVC-cardiomyopathy. The mean % total LGE-scar area was 5%, ranging between 1.3 and 11%. The distribution of LGE was predominantly mid-myocardial in 50% cases, epicardial in 25% of the cases, transmural in 17% of the cases and subendocardial in 8% of the cases. Septal LGE was detected in 67% of the patients, free wall in 25% of the patients and combined (septal and free wall) in 8% of the patients.

**Table 2:** Description of variables analysed from cardiac MRI and electroanatomical mapping

CMR variables	Frequency (proportion)	EAM variables	Frequency (proportion)
Mean LV EDVi	93.3±26.3	Mean number of data points	1046.7±897.1
Mean LV ESVi	60±28.4	Mean LV surface area, sq.cm	143.9±41.8
Mean LV mass, grams	151.7±69.3	Map density, per sq.cm	8.7±8.9
Peak global circumferential strain, %	−11.8±4.5	Mean bipolar voltage, mV	2.9±0.2
Peak global radial strain, %	+22.4±8.7	Mean unipolar voltage, mV	10.3±0.6
Peak global longitudinal strain, %	−11.2±3.8	Mean % area of bipolar LVZ	29.0±22.1
Mean, % segmental abnormality (CS)	75.3±20.0	Mean % area of unipolar LVZ	37.5±21.5
Mean % segmental abnormality (LS)	70.0±15.9	**Location of bipolar/ unipolar LVZ** **(in order of frequency)**	
Mean % segmental abnormality (CS + LS)	54.3±20.4	Basal anteroseptal	12 (63%)
LGE-scar	12 (63%)	Basal inferolateral	10 (53%)
Focal LGE-scar	9 (75%)	Basal inferoseptal	9 (47%)
Multifocal LGE-scar	3 (25%)	Basal anterolateral	8 (42%)
Endocardial/ Mid-myocardial/ Epicardial/ Transmural LGE-scar	1 (8%)/ 6 (50%)/ 3 (25%)/ 2 (17%)	Mid anterior	6 (32%)
Septal/ Free wall/ Combined	8 (67%)/3 (25%)/1 (8%)	Basal anterior	5 (26%)
Mean scar mass, grams	9.3±8.3	Basal inferior	4 (21%)
Mean scar volume, ml	8.9±7.9	Mid anteroseptal	4 (21%)
Mean % LGE-scar area	4.0±3.9		

Values are mean ± SD or n (%)

Abbreviations: *SD*, standard deviation; *CMR*, cardiac magnetic resonance imaging; *EAM*, electroanatomical mapping; *LV*, left ventricle; *CS*, circumferential strain; *LS*, longitudinal strain; *LGE*, late gadolinium enhancement; *bipolar LVZ*, low voltage zone <1.5 mV; *unipolar LVZ*, low voltage zone <8.3 mV

Bipolar LVZ was detected in 12 (63%) patients and unipolar LVZ was detected in all the 19 patients (100%). Mean percentage area of bipolar LVZ was 29±22%. Mean percentage area of unipolar LVZ was 37.5±22.5%. The most common locations of electrical LVZ were basal anteroseptal (63%) followed by basal inferolateral (53%), basal anterolateral (42%), and mid anterior (32%) segments. The segmental values of circumferential strain, longitudinal strain, radial strain, LGE-scar, mean bipolar voltage, mean unipolar voltage and percentage area of bipolar and unipolar LVZ are presented in the *Supplementary Table* S1.

### Correlation between myocardial strain, LGE-scar by CMR and low voltage zones by EAM (Supplementary Table S2; Fig. 1)

Moderate correlation (*p* = 0.07) was noted between % unipolar LVZ and global circumferential strain (*r* = +0.4), and between % unipolar LVZ and global longitudinal strain (*r* = +0.4). Percentage segmental abnormalities detected with longitudinal strain had significant correlation with % area of bipolar LVZ (*r* = +0.5; *p* = 0.03). Percentage segmental abnormalities detected with a combined circumferential and longitudinal strain had significant correlation with % area of unipolar LVZ (*r* = +0.5; *p* = 0.02). Percentage area of LGE-scar showed insignificant correlation with both % area of bipolar LVZ (*r* = +0.2; *p* = 0.2) and % area of unipolar LVZ (*r* = +0.3; *p* = 0.2).

**Fig. 1:**
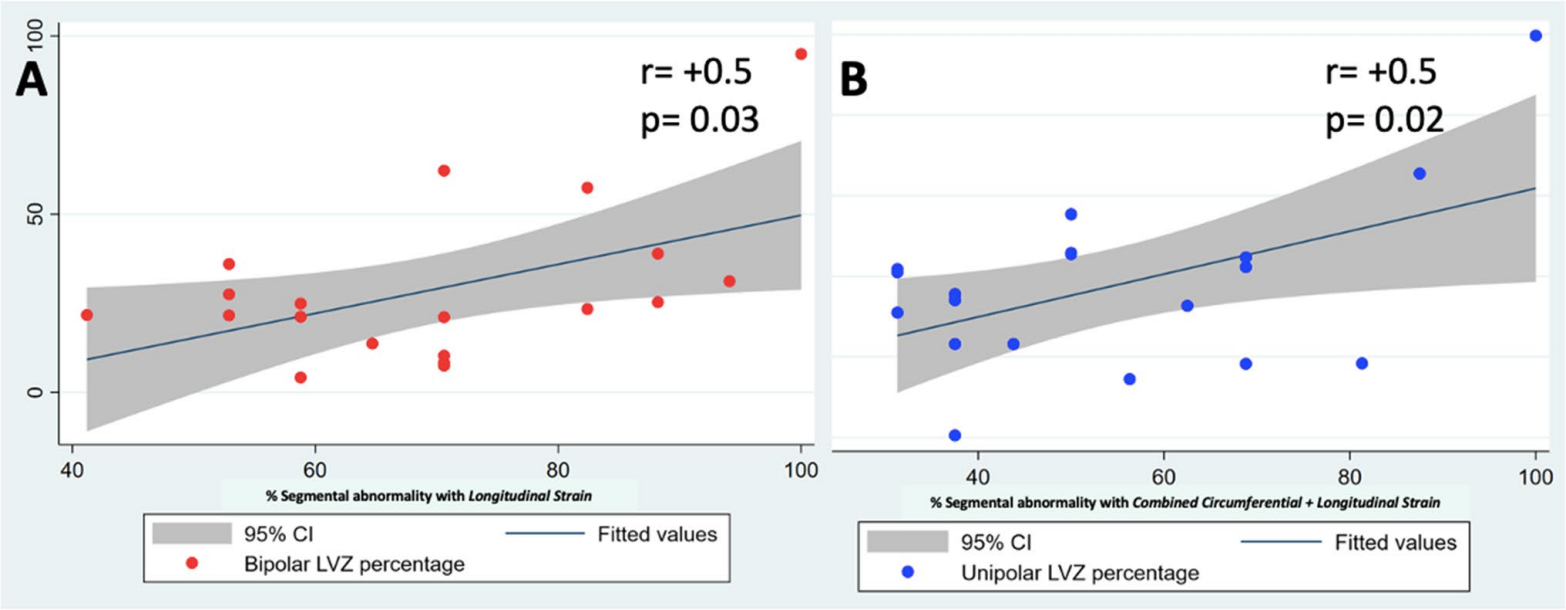
Panel **A** shows the correlation between percentage abnormalities detected with longitudinal strain and percentage area of bipolar low voltage zone (LVZ); Panel **B** shows the correlation between percentage abnormalities detected with combined circumferential + longitudinal strain and percentage area of unipolar low voltage zone (LVZ)

The linear regression analysis revealed a positive relationship between global circumferential strain and global longitudinal strain with % area of unipolar LVZ — one unit increase in global circumferential strain increased the % area of unipolar LVZ by 2.09 (*p* = 0.07) and one unit increase in global longitudinal strain increased % area of unipolar LVZ by 2.49 (*p* = 0.06). However, there was no strong relationship between global circumferential strain and global longitudinal strain with % area of bipolar LVZ.

### Localization of segmental abnormalities with myocardial strain (Table 3, Figs. 2 and 3)

The concordance rate between LGE-scar and bipolar LVZ was 50%. The concordance rate between LGE-scar and electrical abnormalities detected by either bipolar or unipolar LVZ was 63%. Segmental abnormalities of peak circumferential strain had higher concordance rates than LGE-scar, with bipolar LVZ (75%). Similarly, segmental abnormalities of peak circumferential strain had concordance rate of 79% with electrical abnormalities detected by either bipolar or unipolar LVZ. Segmental abnormalities of peak longitudinal strain had better concordance rates than LGE-scar and peak circumferential strain, with bipolar LVZ (92%) and with electrical abnormalities detected by either bipolar or unipolar LVZ (95%). While accounting for combined abnormalities in segmental circumferential and longitudinal strain, the concordance rate was 83% with segmental bipolar LVZ and 89% with segmental bipolar or unipolar LVZ.

**Table 3:** Concordance rate of CMR variables for localization of segmental EAM-LVZs

CMR variables	Bipolar LVZ (*n* = 12)	Bipolar or unipolar LVZ (*n* = 19)
LGE-scar	6 (50%)	12 (63%)
Peak circumferential strain	9 (75%)	15 (79%)
Peak longitudinal strain	11 (92%)	18 (95%)
Peak circumferential + longitudinal strain	10 (83%)	17 (89%)

Abbreviations: *CMR*, cardiac magnetic resonance imaging; *LGE*, late gadolinium enhancement; *bipolar LVZ*, low voltage zone <1.5 mV; *unipolar LVZ*, low voltage zone <8.3 mV

**Fig. 2:**
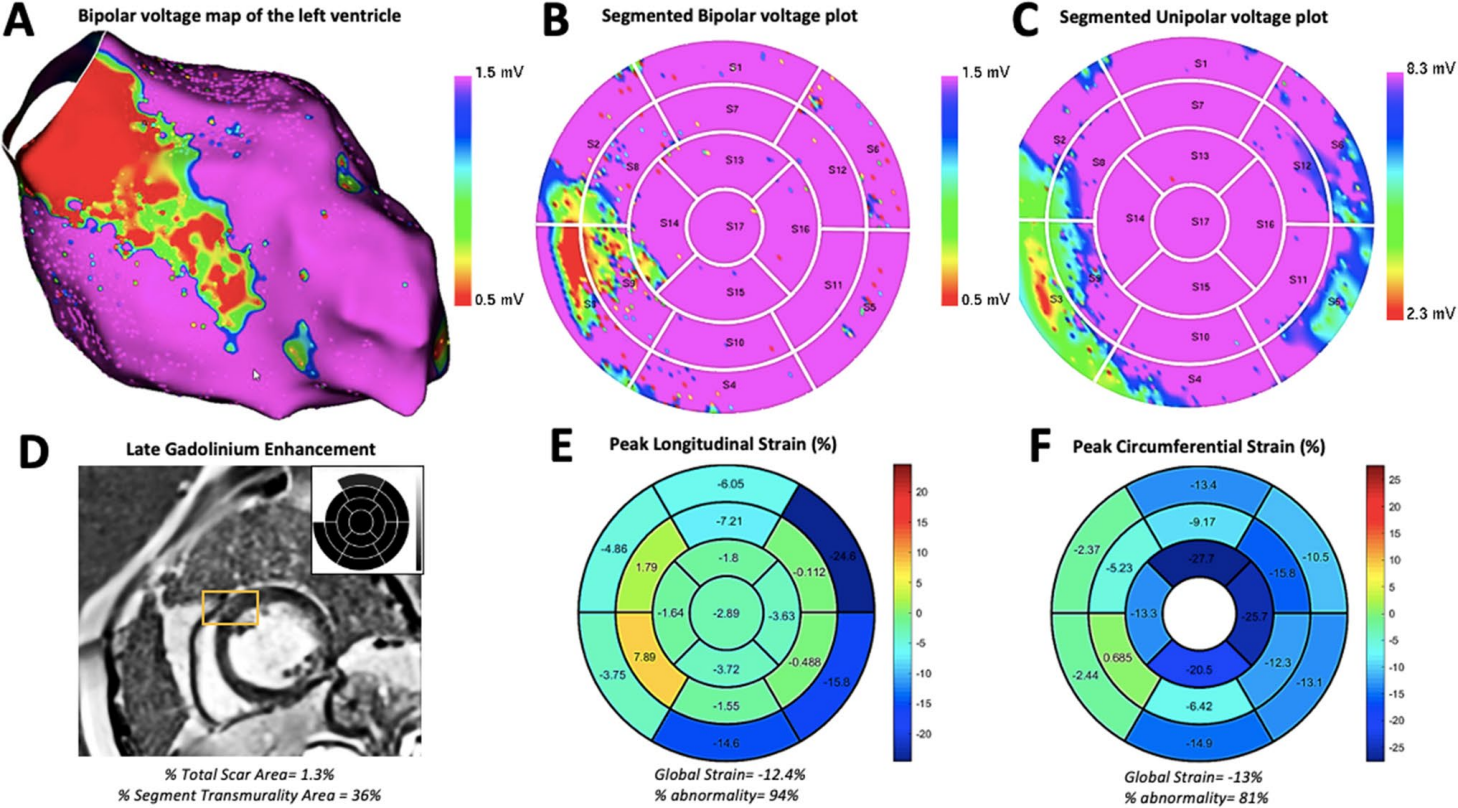
Case demonstration: Panel **A** shows the bipolar voltage map by electroanatomical mapping in a patient of non-ischemic cardiomyopathy with low voltage zones in the superior and inferior basal and mid-interventricular septum; Panels **B** and **C** show the localization of the bipolar and unipolar low voltage zones respectively in the same patient on the segmental polar plots; Panel **D** shows the late gadolinium enhancement (yellow box) in the basal anteroseptum. Also seen in the inset is the segmental representation of LGE in the polar plot; Panels **E** and **F** show the segmental distribution of peak longitudinal and circumferential strain respectively in this patient with abnormalities detected in the entire basal and mid septum

**Fig. 3:**
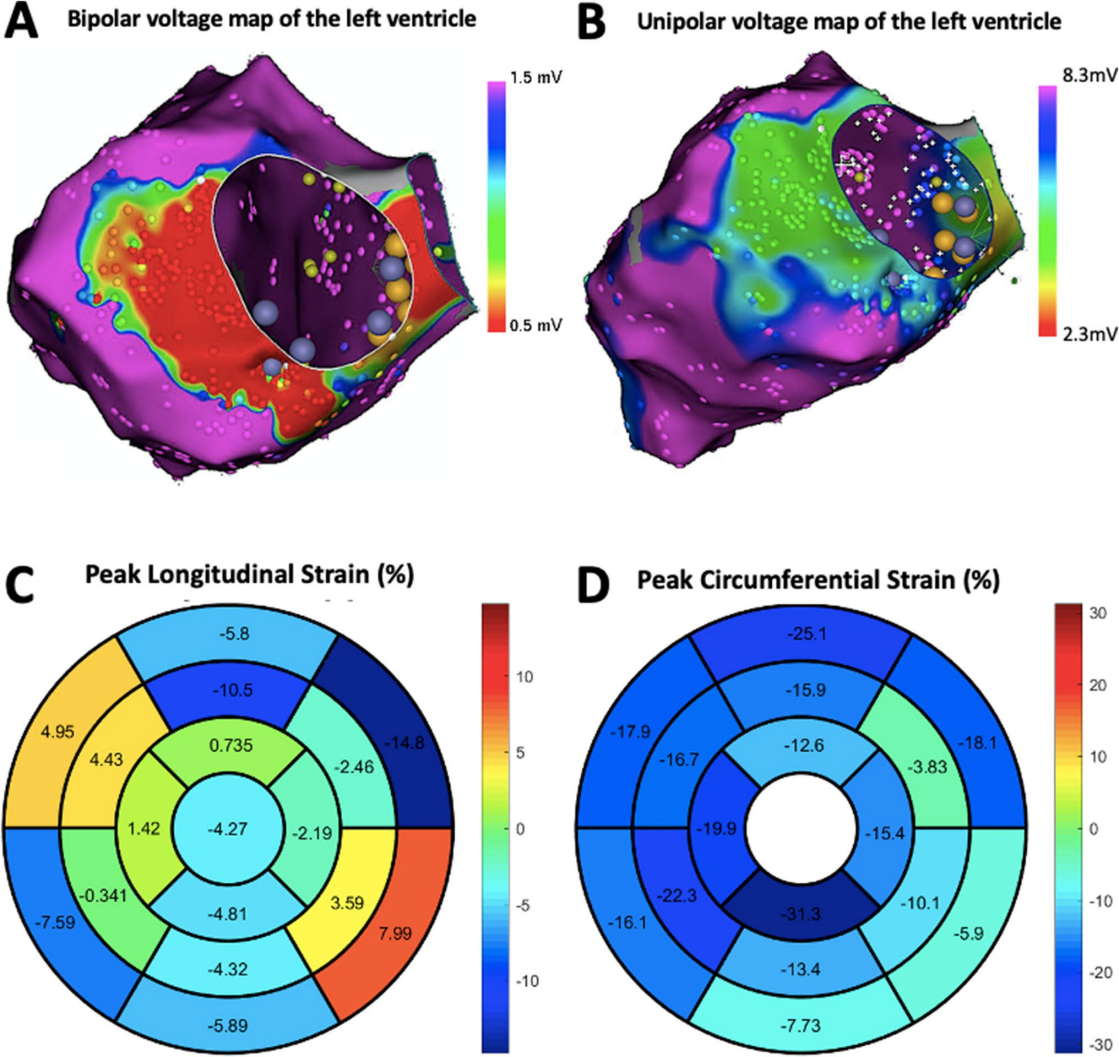
Case demonstration: Panel **A** shows the bipolar voltage map by electroanatomical mapping in a patient of non-ischemic cardiomyopathy with low voltage zones in the inferolateral basal left ventricle; Panel **B** shows the unipolar voltage map by electroanatomical mapping in the same patient with low voltage zones in the anterolateral and inferolateral basal left ventricle; Panels **C** and **D** show the polar plot of segmental distribution of peak longitudinal and circumferential strain respectively in this patient which correlates with both the bipolar and unipolar low voltage zones. Cardiac magnetic resonance imaging with late gadolinium enhancement had failed to detect any abnormality in this region

### Clinical outcomes

Induction of VT by invasive electrophysiological study was performed in all the patients. Overall, 3 patients had hemodynamically stable monomorphic VTs, and 13 patients had repetitive non-sustained monomorphic VTs or hemodynamically unstable VTs. The mean number of monomorphic VTs induced per patients was 1.1±0.73. Bipolar and unipolar low voltage zones were detected in all the patients with inducible VT. VT ablation in form of substrate modification of the low voltage zones was performed in 16 patients. Acute success, as defined by non-inducibility of VT, was achieved in all the patients who had induction of VT. There were no major complications. In patients with no history of VT (*n* = 8), 5 patients had inducible VT. In these, 5 patients with inducible VT, but with no history of VT, only 2 had detectable LGE-scar, while all these patients had abnormal CMR circumferential and longitudinal strain.

The mean follow-up period was 14±3 months. There were no deaths reported in the cohort. Device therapies were noted in 32% of the patients. Patients with VT had higher % area of unipolar LVZ (47±7%) than those without VT (27±5%; p=0.05). There was no significant difference in the means of other variables in patients with and without VT.

## Discussion

The salient findings of our study are [1] Global circumferential and longitudinal strain has moderate correlation with percentage area of bipolar and unipolar LVZs; [2] Percentage segmental abnormality detected with combined circumferential and longitudinal strain has good correlation with percentage area of unipolar LVZs; [3] Localization of abnormalities with CMR-FT strain resulted in better concordance rates of 89% with a composite of circumferential and longitudinal strain compared to only 63% with LGE, for detection of bipolar or unipolar LVZs.

### Basis for investigating beyond LGE in NICM

The distribution of fibrosis in NICM is complex. A histological validation of fibrosis patterns in NICM showed patchy and/or diffuse distribution [17]. The interstitial fibrosis predominantly involves the basal septal, and basal inferolateral segments of the LV [18, 19]. The endocardial bipolar voltages are more sensitive to changes in the subendocardium [16]. However, the substrate in NICM is more likely to be in the deeper myocardial layers like mid-myocardium and epicardium which are more likely to be represented by reduction in the unipolar voltages [20]. LGE has been the conventional modality to detect myocardial scarring. While LGE has an excellent diagnostic value in detection of macroscopic scars as in ischemic cardiomyopathy, it is insensitive to pick up microscopic and diffuse distribution of scars as in NICM [21]. Moreover, detection of LGE relies on the visual assessment of regional differences in uptake of gadolinium [3]. At cut-offs of unipolar voltage <8.3 mV, LGE had a low diagnostic value with sensitivity and specificity of 78% and 43% respectively to detect these low voltage zones [22].

The architecture of the LV myocardium is such that the subendocardial and epicardial fibers are longitudinally oriented and the midmyocardial fibers are circumferentially oriented. Considering the more common mid-wall patchy distribution of scar in NICM, circumferential strain is likely to pick up these abnormalities [6]. Longitudinal strain is sensitive to abnormalities in the subendocardium and epicardium [6, 11]. A composite of these strain measures is likely to be more representative of the underlying myocardial abnormalities. In our study, the percentage abnormal myocardium of only longitudinal strain had good correlation with percentage bipolar-LVZ. The composite of circumferential and longitudinal strain had good correlation with percentage unipolar-LVZ.

### Correlation of CMR-strain with LVZs in NICM (Fig. 2)

The highlight of our study is the higher concordance rate of myocardial strain compared to LGE to detect electrical abnormalities. In patients with right ventricle cardiomyopathies, the correlation between LGE and EAM-LVZs was only 48%. In the same study, 91% of patients with percentage area of EAM-LVZ <20% had no LGE, and endomyocardial biopsy was considered a better tool to correlate with EAM-LVZs [4]. LGE may fail to detect any level of fibrosis variably between 31 and 70% of the patients with NICM [3]. In a study on 90 patients with non-ischemic LV ventricular tachycardia/ventricular premature depolarizations, LGE-to-EAM voltage discordance was noted in 36% of the patients [3]. In our study, the concordance rates between LGE-scar and the EAM-LVZs was only 63%. In 80% of the patients, with percentage area bipolar or unipolar LVZ <20%, there was no LGE. In addition, the percentage area of LGE-scar had poor correlation with the percentage bipolar and percentage unipolar LVZs.

### Methods to measure myocardial deformation

Myocardial strain analysis can be performed by speckle-tracking echocardiography (STE) as well. In a recent study, endocardial longitudinal strain by STE correlated strongly with percentage bipolar low voltage zone and mid-myocardial longitudinal strain correlated strongly with percentage unipolar low voltage zone. In the same study, the concordance rate for regional abnormalities of strain with the LVZs was 75% [11]. As myocardial contraction occurs circumferentially and radially as well, it is likely that circumferential strain, which was not measured in the study, would have improved the concordance rates. While myocardial strain measured by both STE and CMR-FT have good agreement, the reproducibility of CMR-strain is better owing to the high intrinsic contrast and better delineation of blood-endocardial tissue borders [6]. Myocardial strain can also be measured by tissue tagging methods like fast strain–encoded MR (fast-SENC). Fast-SENC has recently been investigated in a large cohort of patients with heart failure and has shown to be predictive of poorer clinical outcomes like death and heart failure hospitalisations [7]. In our study, strain analysis was performed by CMR-FT. The advantage of this technique is that this can be applied to standard cine exams retrospectively and does not require additional time-consuming sequences (as for CMR tagging) [10]. Also, myocardial strain by CMR-FT has reasonable agreement with tissue tagging methods [13].

Alternative CMR diagnostic tools, such as T1 mapping and ECV estimation, are being explored to see whether they better correlate with electrical abnormalities in NICM. In a study on 36 patients with dilated cardiomyopathy, native T1 mapping values and ECV estimation had strong correlation with the collagen fraction noted in the biopsy and was better than LGE in detection of diffuse microscopic fibrosis [23]. In another study with 50 NICM patients with negative LGE, there was a significant inverse correlation between T1 values and the extent of both bipolar and unipolar low voltage areas [23].

In a large cohort of patients with NICM, the presence of LGE and epicardial or transmural distribution of LGE were significant predictors of ventricular arrhythmic events across different strata of LVEF [2]. Though our study was not adequately powered to detect ventricular arrhythmia events and all-cause mortality, large scale studies looking at CMR parameters beyond LGE like CMR strain and T1 mapping values are necessary to ascertain the prognostic significance of these variables [21].

### Limitations

The results of the study should be interpreted in the context of the relatively small sample size of the study and small number of events over follow-up. The cut-off of −17% applied for segmental strain abnormalities was the same as for global strain values, however this needs large scale validation. CMR analysis might not be accurate in patients with bundle branch blocks (5 patients) and high PVC burden (4 patients), though this number was small in this study. This study did not test strain measurements by tissue tagging methods, such as Fast-SENC strain. CMR-FT, despite its distinct advantages, is subject to through-plane motion artefacts. This study did not test other CMR measures like T1 mapping values, assessment of ECV and perfusion scores. Further studies are needed to determine whether these investigations can complement LGE in the risk stratification for sudden cardiac death. Since the introduction of 8.3 mV as the cut-off for defining unipolar low voltage zones, a lot of studies have retained this value. We have also used the same cut-off for defining the unipolar low voltage zones. However, these cut-off values are likely to change, while using high-density mapping with multi-electrode catheters.

## Conclusion

Our study concludes that abnormal myocardial strain detected by CMR-FT method is more closely related to electrical abnormalities, than the conventional LGE detected by CMR. Localization of low voltage zones with CMR-strain has better concordance than LGE. Thus, CMR-strain can inform the operator about specific regions of substrate abnormalities during a VT ablation procedure, especially in the absence of LGE scar. Our study emphasizes the need to look beyond LGE detected by CMR with novel parameters such as CMR-strain while correlating with the LV electrical abnormalities.

### Clinical perspectives

#### Clinical competency

In patients with nonischemic cardiomyopathy, late gadolinium enhancement detected by CMR, while having important prognostic implications, has a poor concordance rate with electrical abnormalities detected by electro-anatomical mapping studies. Alternate measures like T1 mapping values, extracellular volume estimation and myocardial strain are being evaluated to better correlate with low voltage regions of the left ventricle.

#### Translational outlook

Myocardial strain picked up by CMR-feature tracking has good correlation with unipolar low voltage zones. The strain parameters can also be used to detect the segmental abnormalities with concordance rates better than late gadolinium enhancement. This is important for the operator to understand the underlying abnormalities while attempting ablation of the substrate.

### Supplementary information

Supplementary file 1 **Supplemental Table S1**: Segmental values of measured parameters from cardiac MRI and Electroanatomical mapping, **Supplementary Table S2:** Bivariate correlation between the measured parameters
