# Supplementary material for: Correlation of myocardial strain by CMR-feature tracking with substrate abnormalities detected by electro-anatomical mapping in patients with nonischemic cardiomyopathy
Source: J Interv Card Electrophysiol. 2023 May 2;66(9):2113–23. doi: 10.1007/s10840-023-01553-5 (PMC10694091; doi:10.1007/s10840-023-01553-5)
Supplement: Supplementary file 1 — Supplemental Table S1: Segmental values of measured parameters from cardiac MRI and Electroanatomical mapping, Supplementary Table S2: Bivariate correlation between the measured parameters [file 10840_2023_1553_MOESM1_ESM.docx]

**Supplemental Table S1: Segmental values of measured parameters from cardiac MRI and Electroanatomical mapping**

| **Measures** | **S1** | **S2** | **S3** | **S4** | **S5** | **S6** | **S7** | **S8** | **S9** | **S10** | **S11** | **S12** | **S13** | **S14** | **S15** | **S16** | **S17** |
| --- | --- | --- | --- | --- | --- | --- | --- | --- | --- | --- | --- | --- | --- | --- | --- | --- | --- |
| **Mean circumferential strain (%)** | -13.6±  6.1 | -10.4±  7.5 | -8.8±  7.2 | -11.9±  5.0 | -13.9±  8.2 | -14.6±  6.8 | -11.8±  6.0 | -9.6±  10.5 | -9.2±  7.8 | -8.6±  5.6 | -10.7±  4.3 | -8.6±  5.3 | -11.3±  6.8 | -14.2±  12.0 | -17.3±  10.0 | -10.8±  7.0 | NA |
| **Mean radial strain (%)** | 17.1±  20.8 | 4.0±  11.3 | 4.9±  8.6 | 24.5±  13.8 | 25.5±  13.2 | 31.2±  18.7 | 30.9±  14.7 | 18.4±  19.3 | 23.2±  17.4 | 27.1±  14.7 | 27.2±  13.1 | 31.4±  12.8 | 25.3±  15.5 | 15.5±  17.3 | 23.8±  17.5 | 25.2±  15.7 | NA |
| **Median longitudinal strain (%)** | -13.6±7.5 | -15.5±9.6 | -19.8±9.1 | -21.1±7.1 | -25.6±12.1 | -23.5±10.3 | -12.7±5.5 | -6.0±8.8 | -1.1±6.1 | -5.3±7.1 | -9.8±8.2 | -10.9±7.8 | -8.9±5.2 | -8.3±6.7 | -4.9±4.8 | -4.2±4.4 | -10.4±7.4 |
| **% LGE-scar area** | 10.3±  19.5 | 16.8±  22.5 | 6.2±  13.5 | 7.6±  14.0 | 12.2±  21.3 | 6.3±  14.2 | 4.0±9.6 | 3.0±6.9 | 3.4±6.1 | 7.9±11.3 | 7.1±12.1 | 5.0±11.0 | 0.4±1.8 | 0.9±3.3 | 2.8±6.1 | 1.1±3.4 | 0.0±0.1 |
| **Mean bipolar voltage** | 2.9 ±1.2 | 2.5±1.0 | 2.5±1.0 | 2.7±0.9 | 2.6±1.0 | 2.8±1.1 | 3.0±1.2 | 3.1±1.3 | 3.2±1.0 | 3.6±1.6 | 3.1±1.1 | 3.2±1.4 | 2.9±1.3 | 3.2±1.3 | 3.4±1.7 | 3.0±1.5 | 3.2±1.7 |
| **Mean unipolar voltage** | 9.1±3.1 | 7.5±3.1 | 8.6±3.2 | 8.9±3.2 | 8.9±2.4 | 9.8±2.7 | 12.1±3.8 | 12.0±4.0 | 12.6±3.6 | 12.3±3.4 | 11.4±2.5 | 12.1±3.0 | 12.1±3.9 | 12.2±3.5 | 12.6±3.3 | 12.5±3.7 | 11.6±3.6 |
| **% area Bipolar LVZ** | 28.2±29.6 | 38.6±32.5 | 35.8±29.9 | 29.0±27.8 | 32.5±30.1 | 30.7±30.1 | 24.9±29.2 | 27.2±27.4 | 19.9±26.4 | 17.9±25.5 | 17.7±26.3 | 19.9±21.6 | 25.5±28.1 | 18.6±29.7 | 19.4±27.5 | 28.9±31.0 | 22.0±27.1 |
| **% area Unipolar LVZ** | 49.1±27.5 | 66.8±32.7 | 56.6±33.1 | 57.0±34.9 | 48.6±32.2 | 40.3±31.0 | 18.2±30.5 | 21.8±32.1 | 19.7±33.0 | 21.4±29.5 | 16.5±28.0 | 15.1±26.6 | 18.6±34.2 | 18.0±34.6 | 13.4±26.7 | 14.0±28.7 | 21.2±34.6 |
| Values are mean ± SD or n (%); S1- S17 based on American Heart Association standardised myocardial segmentation  Abbreviations: SD- standard deviation, CMR- Cardiac Magnetic Resonance imaging, EAM- electroanatomical mapping, LV- left ventricle, CS- circumferential strain, LS- longitudinal strain, LGE- late gadolinium enhancement, bipolar LVZ- low voltage zone <1.5mV, unipolar LVZ- low voltage zone <8.3mV | | | | | | | | | | | | | | | | | |

**Supplementary Table S2: Bivariate correlation between the measured parameters**

|  | LVEF | Global CS | Global LS | % segmental abnormality with CS | % segmental abnormality with LS | % segmental abnormality with CS+LS | % area of LGE-scar | % area of Bipolar LVZ | % area of Unipolar LVZ |
| --- | --- | --- | --- | --- | --- | --- | --- | --- | --- |
| LVEF |  | -0.8 * | -0.6 * | -0.7* | -0.3 | -0.7 * | -0.1 | -0.05 | -0.2 |
| Global CS | -0.8 * |  | +0.6 * | +0.9 * | +0.2 | +0.8 * | +0.08 | +0.2 | +0.4 $ |
| Global LS | -0.5 * | +0.6 * |  | +0.3 | +0.7 * | +0.7 * | +0.3 | +0.2 | +0.4 $ |
| % segmental abnormality with CS | -0.7 * | +0.9 * | +0.3 |  | +0.1 | +0.7 * | +0.06 | +0.05 | +0.3 |
| % segmental abnormality with LS | -0.3 | +0.2 | +0.7 * | +0.1 |  | +0.7 * | +0.06 | +0.5 * | +0.3 |
| % segmental abnormality with CS+LS | -0.7 * | +0.8 * | +0.7 * | +0.7 * | +0.7 * |  | +0.01 | +0.2 | +0.5 * |
| % area of LGE-scar | -0.1 | +0.08 | +0.3 | +0.06 | +0.06 | +0.06 |  | +0.2 | +0.3 |
| % area of Bipolar LVZ | -0.04 | +0.2 | +0.2 | +0.05 | +0.5 * | +0.2 | +0.2 |  | +0.5 |
| % area of Unipolar LVZ | -0.2 | +0.4 $ | +0.4 $ | +0.3 | +0.3 | +0.5 * | +0.3 | +0.5 |  |
| Values are correlation coefficients expressed in the range from -1 to +1; Values marked with * have significance at p value <0.05; Values marked with $ have significance at p value <0.10  Abbreviations: CS- circumferential strain, LS- longitudinal strain, LGE- late gadolinium enhancement, bipolar LVZ- low voltage zone <1.5mV, unipolar LVZ- low voltage zone <8.3mV | | | | | | | | | |
